# Behaviour change interventions for responsible antimicrobial use on farms

**DOI:** 10.1186/s13620-023-00236-x

**Published:** 2023-04-03

**Authors:** Áine Regan, Alison Burrell, Claire McKernan, Hannah Martin, Tony Benson, Conor McAloon, Edgar Garcia Manzanilla, Moira Dean

**Affiliations:** 1 Department of Agri-Food Business & Spatial Analysis, Teagasc Mellows Campus, Athenry, H65 R718 Co. Galway Ireland; 2 Animal Health Ireland, 2 – 5 The Archways, Carrick-On-Shannon, N41 WN27 Co.Leitrim Ireland; 3 Institute for Global Food Security, Biological Sciences, Queen’s University Belfast, 19 Chlorine Gardens, Belfast, BT9 5DL Ireland; 4 School of Veterinary Medicine, University College Dublin, Belfield, Dublin 4 Ireland; 5 Pig Development Department, Teagasc Animal and Grassland Research and Innovation Centre, Moorepark, Fermoy, Co. Cork Ireland

**Keywords:** Antimicrobial resistance, Antibiotic resistance, Behaviour change, Behavioural science, Co-design

## Abstract

**Background:**

In the coming years, major governance changes in the form of policy directives and regulations will catalyse major top-down change with respect to animal health on European farms in an effort to combat the OneHealth threat of antimicrobial resistance. This top-down approach must be met with bottom-up strategies to ensure target actors (namely, farmers and vets) are supported and motivated to change their practices, thus, avoiding unintended consequences of forced change. Although much behavioural research has explored the factors influencing antimicrobial practices on farms, a gap exists translating these findings into evidence-based behaviour change interventions that can be put into practice. The current study aims to fill this gap. It provides insights into identifying, understanding, and changing the behaviours of farmers and veterinarians with respect to the responsible use of antimicrobials in farming.

**Results:**

Through an inter-disciplinary and multi-actor approach, the study combines scientific knowledge from the behavioural sciences and animal health sciences, coupled with tacit knowledge from a co-design, participatory approach to recommend seven behaviour change interventions that can help to support good practices amongst farmers and vets, with respect to animal health, and reduce the use of antimicrobials on farms. The behaviour change interventions include message framing; OneHealth awareness campaign; specialised communications training; on-farm visual prompts and tools; social support strategies (for both farmers and vets); and antimicrobial use monitoring. The study details each intervention with respect to their evidence base and scientific concept, grounded in behavioural science, along with stakeholder feedback on design and delivery of the interventions.

**Conclusions:**

These behaviour change interventions can be taken, adapted, and put into practice by the agri-food community to support good animal health practices and responsible antimicrobial use on farms.

**Supplementary Information:**

The online version contains supplementary material available at 10.1186/s13620-023-00236-x.

## Background

With the growing OneHealth threat of antimicrobial resistance (AMR), antimicrobial use (AMU) on farms has increasingly been the focus of governance and policy changes. The European Commission [1] has set a 2030 target of reducing sales of antimicrobials for farmed animals and in aquaculture by 50%. As of 2022, European Union regulations (2019/4; 2019/6) will dictate the use of veterinary medicinal products and medicated feed. This will mean significant changes for farmers and vets with regard to how and when antimicrobials can be used.

AMR is an issue of key strategic concern on the island of Ireland. Ireland’s second One Health Action Plan on Antimicrobial Resistance 2021–2025 (iNAP2) outlines the actions taken by many stakeholders in the animal health sector in recent years to reduce AMU but also highlights that significant quantities of antimicrobials are still in use and call for further work to increase awareness and change amongst stakeholders [2]. Covering Northern Ireland, the UK’s five-year national action plan ‘tackling antimicrobial resistance 2019–2024’ also outlines a number of urgent actions needed at veterinarian and farm level to reduce antimicrobial usage, highlighting the key role that behavioural research will play in determining best strategies to achieve this [3].

Recent research has highlighted the significant challenge in changing the routine and ingrained way key actors (farmers and vets) have used antimicrobials for many years [4]. Changing behaviour has been identified as a crucial component by the World Health Organisation and the European Union to support responsible AMU. Much behavioural research has been carried out with farmers and vets exploring the factors influencing antimicrobial practices on farms [4–6]. However, a gap exists in the literature to translate these findings into evidence-based behaviour change interventions that can be put into practice on farms. Behavioural science can support a systematic and evidence-based approach to the selection and design of effective behaviour change interventions. Research observes that policy initiatives which attempt to change behaviour are frequently chosen based on the ISLAGIATT principle, an acronym for ‘it seemed like a good idea at the time’ [7]. This refers to the tendency to decide on the type and content of an intervention, prior to fully understanding what behaviours the intervention needs to change, and how exactly they will change it. Behavioural science frameworks, in contrast, facilitate an approach which draws on theory and best evidence for behaviour change intervention design, ensuring a thorough understanding of the behaviours that need to be addressed, and a targeting of the factors driving that behaviour [8]. These frameworks integrate behaviour change techniques [9] as the active ingredients in interventions, which are strategies that have been proven in psychological research to affect behaviour change at the individual level.

Top-down interventions for behaviour change such as legislation and penalties for non-compliance can result in improvements to farm practices and animal welfare [10]. However, a top-down approach can also be perceived as leaving implicated actors with little control or power, which can lead to impacts on well-being [11]. It can also lead to unintended consequences like a disconnection from what is considered good farm practice [12] or a change in AMU rather than a reduction [13]. Evidence from behavioural science suggests interventions that combine restrictive measures with enabling measures (e.g., education & training, restructuring the environment, communications & messaging, incentives, and intervention targeting) are more successful [8]. Restrictive measures may not target those factors that are likely to bring about motivation to change one’s behaviour. For example, new legislation may mean that a farmer or vet knows they have to change their behaviour, but they may not see the need or value to them personally of changing their behaviour [4].

The socio-ecological framework is a behavioural model that acknowledges behaviour as influenced by a range of inter-related factors at the intra-personal, inter-personal, community, and societal level [14]. By acknowledging the complexity of these factors influencing behaviour change, a socio-ecological approach goes beyond examining change from a purely regulatory, “top-down” perspective. Adopting a social ecological approach, which comprises both top-down and bottom-up interventions, can lead to more meaningful, sustainable behaviour change [14]. In conjunction with the necessary changes at public policy level, this approach involves also targeting an individual’s own knowledge, attitudes and skills within their social environment including family, social network, organisations, communities and wider society [15]. ‘Responsible AMU’ is a multi-faceted concept and does not involve any one single behaviour or practice change. Instead, there is a need to bring about a cultural shift in how farmers and vets manage animal health on farms, in particular, favouring a more proactive, rather than reactive, model for managing animal health (e.g. through improved hygiene, vaccines, biosecurity etc.).

Employing a participatory approach to co-design behaviour change intervention options enhances their suitability and effectiveness in practical application [16]; particularly where animal health farming practices are concerned [10, 17, 18]. The aim of the current study was to use an evidence-based and co-designed intervention development process to identify the building blocks for specific behaviour change interventions that could be adopted and further developed, piloted, and used by different actors across the agri-food setting to help reduce AMU on farms on the island of Ireland.

## Methods

### Approach

**Fig. 1:**
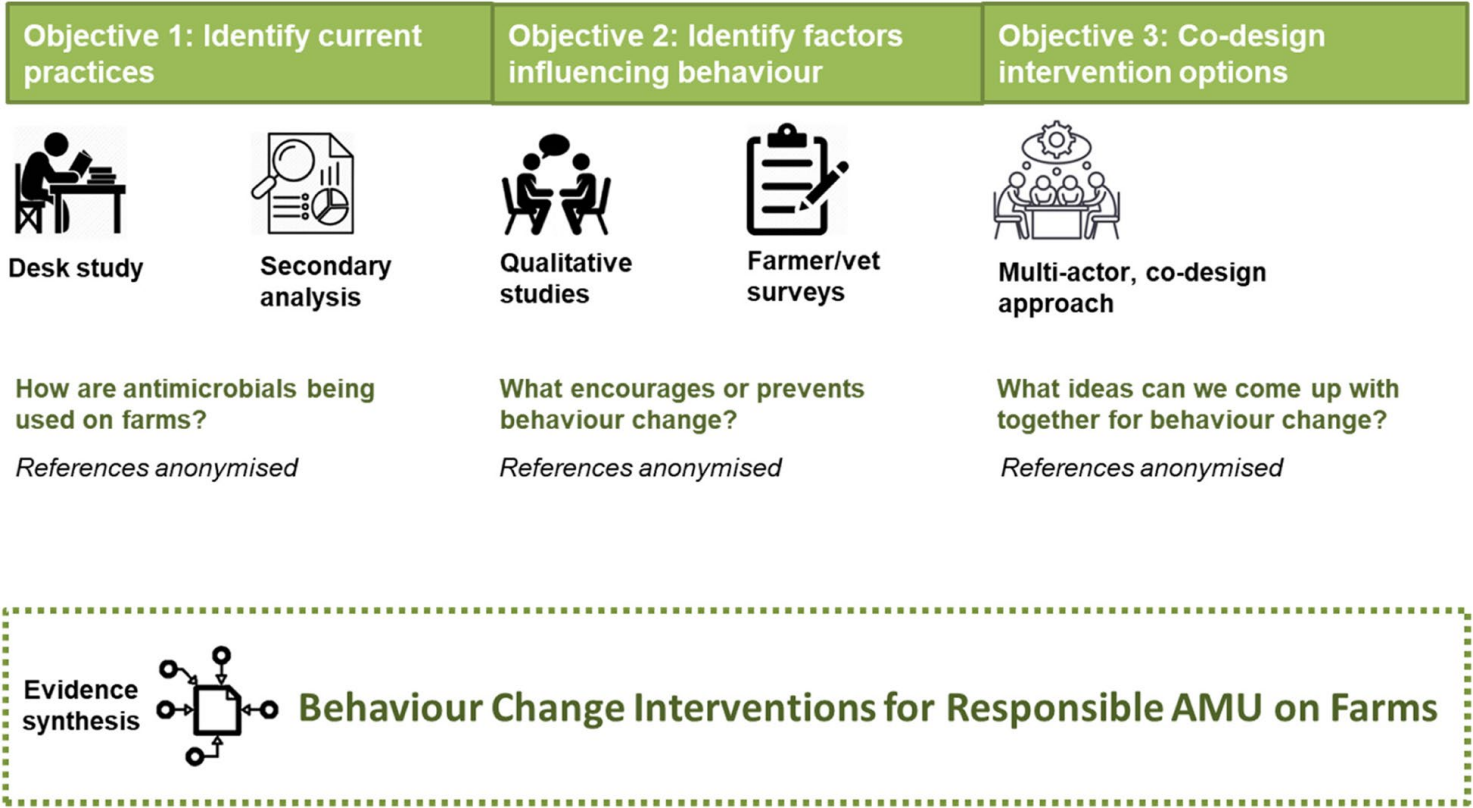
Process for the development of evidence-based, co-designed behaviour change interventions for responsible AMU on farms

As shown in Fig. 1, behaviour change interventions were developed from (1) an inter-disciplinary research base, previously documented and published [4, 19–22]; and (2) a participatory co-design approach, documented in the current study.

The participatory approach was conceptualised as providing rigorous methods for engaging a variety of actors to ensure that the people who must make the changes are involved in the process of designing the behaviour change interventions. Participatory Learning and Action (PLA) methodology was used to facilitate this engagement, with tools such as stakeholder mapping and participatory workshops adapted from previous research [10, 16–18].

### Stakeholder engagement: sampling strategy

In March 2019, a face-to-face participatory workshop with the scientific team and stakeholder advisory board (*n* = 12) served as a starting point activity for the sampling strategy and to identify relevant actors with an interest and stake in AMU and AMR on the island of Ireland. Workshop participants took part in brainstorming activities. Stakeholder maps (Additional File 1) were produced that highlighted the many different types of expertise, knowledge, and perspectives relevant to developing behaviour change interventions. The maps were used as a basis for the selection of stakeholders to invite to participate in subsequent co-design activities, broadening the intervention design process to a wider, and more inclusive, range of stakeholders.

The selection of stakeholders for engagement aimed to be as inclusive as possible with respect to stakeholder type, gender, and region to achieve diverse and reflexive thinking throughout intervention planning. Purposive sampling strategies were used to select key and relevant stakeholders for engagement for each co-design activity. In total, 70 stakeholders representing a wide and diverse range of experience, values, knowledge and expertise provided formal input during the planning, development and evaluation of the intervention recommendations. The gender breakdown was 43 males and 27 females from the farming community (*n* = 16), veterinary sector (*n* = 13), farm advisory & education (*n* = 12), research & education (*n* = 15), government & regulators (*n* = 10), and industry (*n* = 4). Some stakeholders engaged with the project at different points throughout the project, resulting in a total number of 92 separate stakeholder engagements during the intervention development process (Table 1).

**Table 1:** Co-design process for development of AMU behaviour change interventions

Month	Co-design stage	Method	Participants	Input
Mar ‘19	Identifying stakeholders	Face-to-face workshop (*n* = 12)	Stakeholder advisory board & research team	Stakeholder map, broadening range and type of stakeholders for co-design process
Aug ‘20	Co-designing ideas	One-to-one stakeholder consults via telephone/Zoom (*n* = 2)	Lecturers training student vets	Insights on successful training-based strategies for vets and student vets
Sep’20	Co-designing ideas	Small group stakeholder consults via Zoom (*n* = 12)	Animal health experts, farm advisors, farmers, co-op representatives, researchers	Insights on logistics for delivering specialised training, community of practice approach and best approaches for monitoring AMU
Oct’20	Co-designing ideas	One-to-one stakeholder consults via telephone/Zoom (*n* = 2)	Knowledge transfer experts in education and advisory	Insights on training and CPD for farm advisors and logistics of delivering training to farm advisors
Nov’20	Co-designing ideas	One-to-one stakeholder consults via telephone/Zoom (*n* = 10)	Farmers from the pig, dairy, beef, sheep industries	Insights into farmers’ views and understanding of AMU and good antimicrobial stewardship, across different sectors and regions
Mar—May’21	Co-designing ideas	One-to-one stakeholder consults via telephone/Zoom (*n* = 6)	Animal health experts, vet and farm advisor trainers, behavioural scientists, policy makers, vets	Insights on successful training strategies for vets and farm advisors
Mar—May’21	Co-designing ideas	One-to-one stakeholder consults via telephone/Zoom (*n* = 7)	Animal health experts, farmers, vets and farm advisors	Insights on practical, local knowledge of farming sectors and delivering/participating in herd health consults
Mar—May’21	Co-designing ideas	One-to-one stakeholder consults via telephone/Zoom (*n* = 6)	Animal health experts, farmers, vets and farm advisors	Insights on critical control points and ‘gold standard’ hygiene and health management practices in the dairy and pig sectors
Aug – Sep ‘21	Evaluating 7 behaviour change interventions	Online interactive exercise using survey and video software (*n* = 35)	Animal health experts, educators, scientists, policy makers, vets, farmers, industry	User feedback and sense checking; refinement and validation of 7 behaviour change interventions

### Participatory methods

Participatory exercises with stakeholders took place online during the course of August 2020 – September 2021. Given Covid-19 restrictions during this time, in lieu of planned participatory workshops, one-to-one phone calls, smaller online meetings and interactive online software were utilised to engage stakeholders for specific aspects of the intervention co-design process. The extent of each engagement was in-depth; with stakeholder engagement exercises ranging in length from 1 to 3 h and requiring active participation from the stakeholders. Broad directions for intervention options were formulated based on the insights revealed from the interdisciplinary research. Some consults were specifically structured to discuss these ideas with stakeholders while other consults were left deliberately open to facilitate new ideas to emerge. An unstructured approach was used for all consults where ideas were openly exchanged, and free dialogue took place to support idea creation and bottom-up feedback from the participants. Table 1 details all stakeholder engagement co-design activities which took place. Informed consent was obtained prior to each stakeholder engagement.

## Results and discussion

**Fig. 2:**
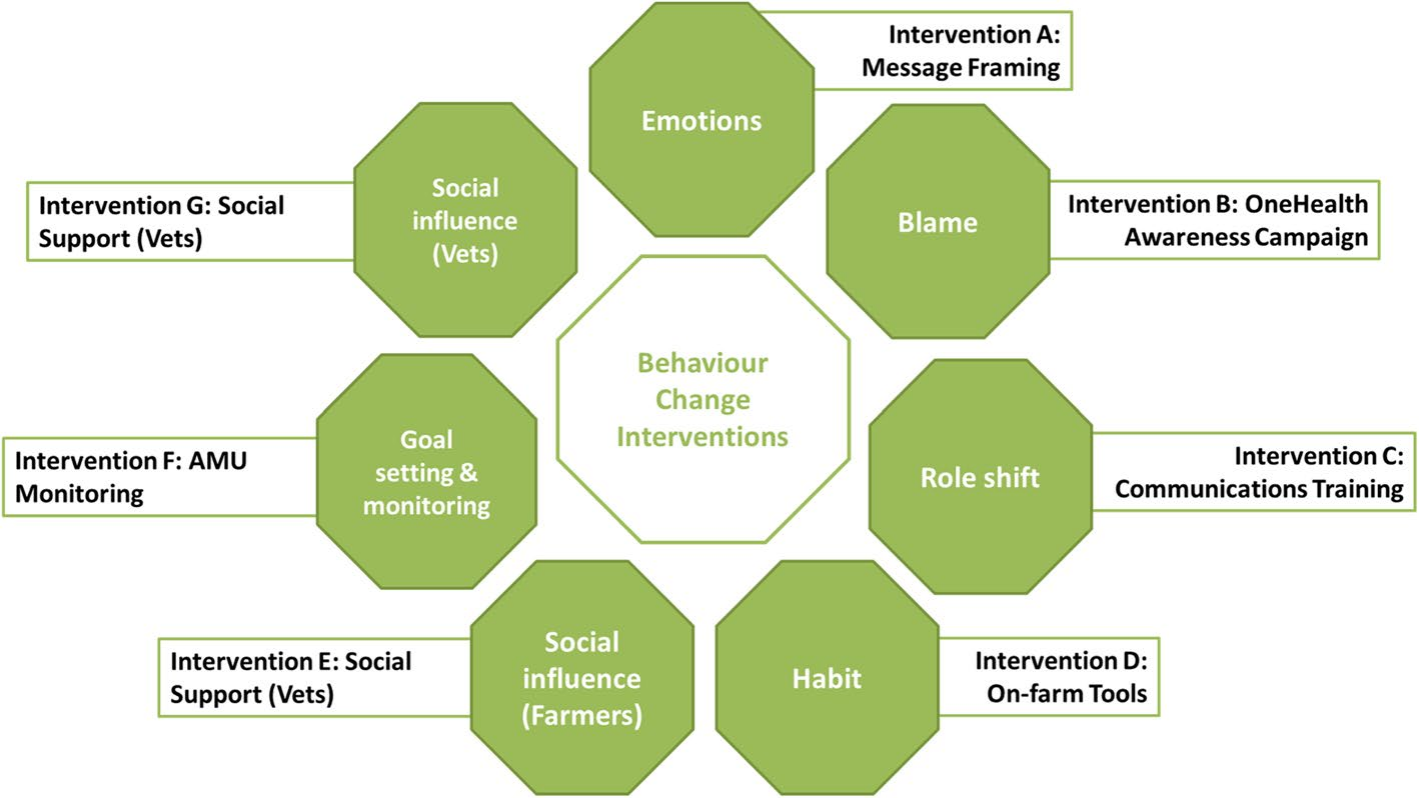
Seven AMU behaviour change interventions selected to target main themes emerging from inter-disciplinary research

Seven behaviour change interventions targeted at responsible AMU on the farm were developed from (1) the inter-disciplinary research base; and (2) the participatory co-design approach (Fig. 1). In a highly iterative process, each intervention targeted a key behavioural finding or theme which emerged from the inter-disciplinary research findings (Fig. 2). The structure of the interventions was further informed by stakeholder contributions evoked through the co-design process.

**Fig. 3:**
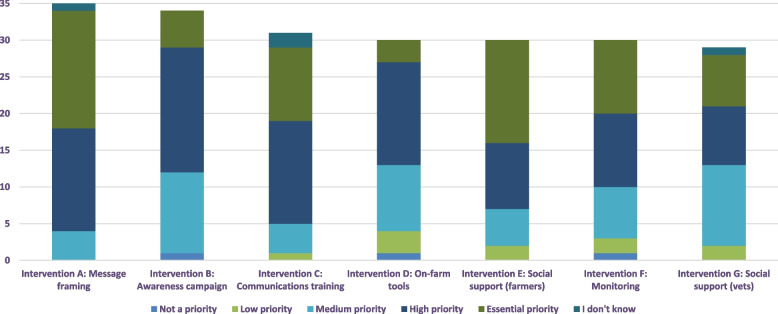
Prioritisation of interventions by stakeholders (n = 35) in online engagement exercise evaluating seven AMU behaviour change interventions. (Note: not all participants provided ratings for every intervention)

In the sections that follow, a more detailed discussion of each intervention is provided along with stakeholder recommendations from the participatory research for design and delivery. A prioritisation of the seven interventions based on the final online interactive exercise with stakeholders provides an indication of community acceptance for each intervention (Fig. 3). All interventions were generally well received. Interventions A (message framing), C (communications training) and D (social support for farmers) received the highest prioritisation across stakeholders.

### Intervention A: reframe the way we talk about AMU and AMR

Empirical research with farmers and vets revealed fears that changing AMU is risky to animal health and an abrupt change could compromise animal welfare and productivity [4, 19, 20]. Vets feared that legislative change would restrict AMU and compromise their ability to treat animals [19]. For farmers, anticipating change induced feelings of uncertainty and stress [19, 20]; conversely, those farmers with high AMR awareness, high self-efficacy and positive emotions were more likely to be ready to change AMU [19]. This suggests that both vets and farmers have strong emotions associated with reducing current AMU on the farms and thus any intervention in this area has to appeal to the emotions as well as cognition. Intervention A aims to target vet and farmer feelings by rigorously considering the language used when communicating to farmers and vets about AMR and making changes to AMU, particularly in the context of new regulations. Intervention A was widely supported: 86% of stakeholders (*n* = 35) completing the online evaluation rated this intervention as a “high” or “essential” priority.

**Fig. 4:**
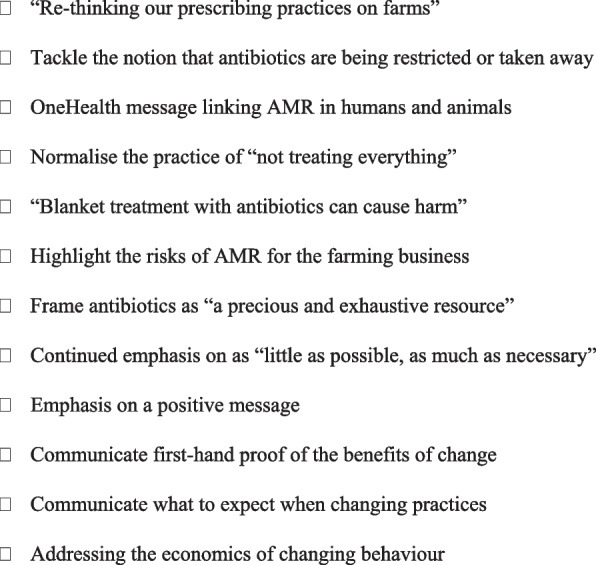
Suggested new ways of talking about AMU and AMR with the farming community

AMR message-framing has been highlighted as an important strategy for informing, motivating and persuading audiences to take action [23]. This intervention is grounded in cognitive reframing/framing and the use of ‘framing principles’ to develop effective messages and communications. Psychologists employ reframing as a therapeutic strategy to change the way that circumstances, experiences, events, thoughts, and/or feelings are viewed in order to allow them to appear more manageable [9]. As our research shows, the changes that may be required for some farms or agricultural sectors under the new AMU legislation may seem overwhelming. Message framing strategies can reframe change as more relevant, manageable and empowering—energising individuals to make changes and protect against feelings of stress or concern, and avoid feeling coerced into change [24]. Examples of framing principles include targeting key values of the audience; using clear, positive and personally relevant language; framing messages positively; addressing uncertainty and risk; and highlighting personal control and actions that can be taken [25]. Specific suggestions emerged from the participatory research for ‘new ways’ of talking about antimicrobials and AMR to help create a culture change around how antimicrobials are viewed and valued. (Fig. 4).

Stakeholders advised that the factual content of all messages be strongly situated in an underlying evidence base; and for messaging to be accompanied by meaningful interventions (e.g., training, support, regulations, and industry changes) that would support safe and sustainable change. Stakeholders cautioned an approach that focused heavily or solely on organisation-led messaging, which could lead to a devolution of responsibility to others and an abdication of action. A message framing approach that was overly simplistic and did not acknowledge existing levels of knowledge and action already taken in the community could backfire. There was strong stakeholder support for a centrally co-ordinated communications strategy for the industry. The formation of a “cross-body communications group” or “working group” with good representation from different organisations could consider how best to apply message-framing principles, agree messaging, and ensure consistency in communications across the industry.

### Intervention B: undertake a OneHealth cross-border AMR awareness campaign

The empirical research with both farmers and vets found that blame for imprudent AMU is likely to be attributed to other stakeholders [4]. Farmers in particular believe antibiotics are used too much in human medicine and other agricultural sectors compared to their own sector [19]. This showed that some farmers and vets readily blame “another” rather than take responsibility and address the problem themselves. These findings highlight the need for an intervention that motivates feelings of collective responsibility amongst farmers and vets for AMR. Intervention B proposes a OneHealth, cross-border AMR awareness campaign whereby responsible organisations could develop a set of consistent and collaborative communication materials (posters, infographics, videos, social media content) that could be used locally in different settings (hospitals, veterinary practices, clinics, educational settings, etc.). Intervention B was moderately supported: 65% of respondents (*n* = 34) in the online evaluation rated this intervention as a “high” or “essential” priority.

This intervention is grounded in research that shows that ‘othering’ and ‘other blaming’ is a common strategy employed when humans seek to attribute blame, particularly relevant to OneHealth crises [26]. Research shows that we often view a problem to be the cause of some group other than our own group, resulting in inaction within our own group [27]. This intervention could help to demonstrate collaboration between different sectors, avoid a culture of blame, ensure consistent and cohesive public messaging on AMR, improve awareness and risk perceptions of AMR in a responsible manner, and ultimately increase motivation of all actors to take small steps for big change: food consumers, farmers, patients, doctors, and vets. This approach has been used previously in the UK where human and animal medical organisations designed a OneHealth poster for use in both vets’ and doctors’ practice waiting rooms, to highlight how responsible use of antibiotics is necessary in both human and veterinary medicines [28].

Although stakeholders were positive about the idea, there was a perceived risk that contrary to the stated objectives of encouraging collective responsibility and action, the juxtaposition of all sectors could create opportunities for comparison and actually lead to increased polarisation where one sector would blame the other for the development and spread of AMR. Stakeholders were also concerned about a devolution of responsibility if actors latched on to that part of the messaging that highlights the problem in other sectors, or they may wait for other sectors to implement change first, leading to a stagnated response by all sectors. Stakeholders were also concerned that an awareness campaign that lacked sufficient detail could be misconstrued by the wider public, and the agri-food sector could come under scrutiny and attack. Emphasis thus needs to be placed on the message of responsible AMU in farming, rather than eliminated AMU, and the continued focus on the actions required in human health. Stakeholders stressed the need for clear language; consistent messages and graphics in both jurisdictions; continuous and widespread messaging; engaging material with practical examples; and, appropriate channels including mainstream media advertising. Stakeholders identified key messages that should be communicated including: (1) the OneHealth message (“*resistance can move between animals and humans*”); (2) that everyone is responsible for acting on AMR (“*we’re all in it together*”); and (3) that AMR is a cross-border issue (“*bacteria know no borders*”). These messages resemble similar narratives from the Covid-19 pandemic. Stakeholders felt the collaborative model and the wide-scale, pervasive nature of the Covid-19 awareness campaign (and the subsequent Covid-19 vaccination campaign) was effective and that lessons could be learned for AMR.

Stakeholders pointed at trust-based challenges in rolling out a OneHealth and cross-border campaign. Different dynamics and cultures surround antibiotic prescribing in animal and human medicine, and thus, it could be difficult to find messaging that would resonate with both sectors. Some cautioned that a cross-border campaign could ‘politicise AMR’ and run into political tensions, mistrust in cross-border messaging, and difficulties in terms of identifying shared objectives. Strong governmental leadership and meaningful collaboration was viewed as essential, as were strong cross-border foundations and relationships that already exist in the area of animal health. Although largely viewed as a governmental-led campaign, stakeholders highlighted that commitment from the key stakeholder groups, including industry, was critical so they would engage with the campaign and ensure a consistent message was being communicated.

### Intervention C: provide specialised communications training for animal health and advisory professionals

The empirical research found that farmers view vets and farm advisors as important and trusted information sources for herd health management [4, 19, 20]. Meanwhile, vets report feeling pressure from clients to prescribe antimicrobials. This demonstrates the difficult relationship between the vet and farmer in the farming process where vets and farm advisors are a trusted source for the farmer until they are not willing to fulfil the farmer’s wishes. This also highlights the need to ensure positive farmer attitudes towards AMU change and avoid farmers feeling coerced [4, 19]. Vets believe open communication with farmers is a vital component to encourage change, are supportive of a ‘role shift’ towards tailored approaches involving herd health visits; and believe training and support to improve communication skills would be helpful [19]. These insights along with a desk review [21] informed Intervention C which proposes specialised communications training to animal health and advisory professionals, to support a transition towards collaborative herd health planning. The intervention was well supported: 77% of respondents in the online evaluation (*n* = 24) rated it as a “high” or “essential” priority.

Stakeholders valued the bottom-up, collaborative nature of this particular approach. There was a strong emphasis on the need for a role shift of vets from reactive to proactive; and also, for a community-of-practice approach that sees vets and farm advisors work collaboratively together with the farmer to move from treatment to prevention in animal health management on farms. Research stresses the importance of this flow of knowledge transfer between farmers and animal health experts, and promotes a multi-actor community to reduce the reliance on antibiotics through developing collaborative herd health plans [29]. For this role to work successfully, vets and farm advisors must be able to deliver technical advice and information successfully. Without the correct communication skills, what may seem like providing good advice and expert opinion may in fact have the opposite effect. Psychological reactance [30] can occur when advice and information is provided to someone by an expert in a top-down, instructive way; rather than having the intended consequence (the person making the necessary changes), it can result in the opposite – the person starts to think of all the reasons not to change and subsequent disengagement with the well-intended advice. In contrast, specialised communication techniques can be used to encourage a collaborative relationship between the professional and their client. Advisors and vets could be trained in these techniques and then put their skills into practice when they carry out consults or appointments with farmers. Possible strategies to explore are a programme to train vets and farm advisors in the use of behaviour change techniques [9, 31] and to train vets in motivational interviewing [32]. These specialised communication techniques have been used across healthcare settings to promote positive behaviour change when speaking with clients. They have been used successfully by professionals in a wide variety of settings such as health care, rehabilitation, public health, social work, dentistry and more recently, motivational interviewing has been successfully used by veterinarians [33].

Stakeholders felt most vets and farm advisors would welcome such training, particularly as it is not currently offered through mainstream education. However, others cautioned that not everyone would be open to such training and if motivation is absent, then vets could react negatively to being offered it. Some resistance from vets and advisors to this type of approach could be encountered, as it is a very different way of doing the job they have known and are comfortable doing. Because of potential motivational issues, it was recommended that the manner in which the communication training and intervention itself is framed and introduced to the vets and advisors would need to be carefully considered. Challenges such as time and cost were also cited as barriers for vets and advisors to take part in the training and to engage in the work involved with collaborative herd health planning. In this respect, participants highlighted the need to ensure sufficient benefit was perceived by those being asked to take part, such as monetary incentives, CPD credits or certification. Stakeholders noted substantial resources would be required to get this type of intervention up and running including those needed for training, but also those needed for co-ordinating herd health planning programmes.

### Intervention D: co-design on-farm tools and prompts

The empirical research found that farmers are most comfortable with a stepwise approach to changing animal health practices, building up confidence in an incremental manner; and identified hygiene practices as an important first step [20]. Vets too highlighted the importance of promoting and supporting alternative behaviours to replace current AMU behaviours [19]. Key hot spot areas for targeting specific problematic AMU behaviours were identified for change in an incremental manner [19, 22]. This highlights the need for starting with achievable small changes that will give confidence to farmers and vets to take the next steps. These findings informed Intervention D which advises to design user-friendly tools and strategies that promote new habit formation through prompts that target small, stepwise and specific but meaningful on-farm changes, such as improved hygiene practices. It was moderately supported, with 57% of respondents (*n* = 17) rating this intervention as a “high” or “essential” priority.

This intervention is grounded in the behavioural science that adding prompts or cues into the environment that clearly explain a desired behaviour can help to promote new habit formation [9]. Involving end-users in the design process of these visual cues helps to ensure the usability of the tools created [18]. Ireland’s Department of Health took a similar behavioural science approach to improving hand washing during the COVID-19 pandemic [34], while a visual feedback approach is also being introduced in Ireland for the management of animal health [35]. Such aids once carefully designed in conjunction with animal health experts, and current good practice guidelines and recommendations can demonstrate the correct actions to take in an accessible manner, can act as good reminders for the farmer, and are useful for encouraging the formation of good habits. Stakeholders felt tools such as visual prompts and aids would be particularly helpful for farm practices that involve multiple actions, resembling ‘checklists’ or standard operating procedures. The ‘on-farm’ and specific nature of this approach bring key AMU actions into the every-day context and lives of the farmer, providing practical, targeted and relevant advice to the farmer about what actions they can take and specific behaviours to target. They would be particularly useful for bigger farms where multiple farm staff are working, and could be used for staff training and to visually demonstrate Standard Operating Procedures (SOPs) and ensure that all farm workers’ (and farm visitors’) behaviours are consistent.

Co-design of the materials was viewed as an important design consideration, with particular reference to engaging with farmers to ensure the material is perceived as useful, relevant and welcomed, rather than a tick-box exercise or a nuisance. Stakeholders cautioned that if the tools are insufficiently clear, they could lead to confusion or adoption of the wrong procedures. For some practices, visual aids would not be appropriate. Others mentioned how such tools could trivialise the issues facing farmers and that farmers could perceive such tools as serving to demean their experience. There was some concern that farmers would not be motivated to pay attention to the tools or that over time their visual impact could wane. Some stakeholders suggested first initiating a strategy to ensure farmer motivation prior to deploying such visual aids. Thereafter, the visual tools could be distributed as useful prompts and supports for them to help enact behaviour change. Stakeholders suggested utilising the visual aids as part of broader interventions which would include elements such as on-farm support (e.g. from vets), reinforcement (e.g. audits) and incentives (e.g. integrate their use into quality assurance or certification schemes). Several stakeholders also made the comment, as with previous intervention ideas, that this intervention would be best implemented as part of a broader strategy that looks to utilise *all* types of behaviour change interventions and provide support from multiple angles.

### Intervention E: encourage peer-to-peer social support and modelling of good farming practices for farmers

The empirical research showed that peers are a trusted source of information [19] and that peer-to-peer learning is judged an effective approach for animal health management [20]. This suggests that peer-to-peer learning is an excellent mode to instigate behaviour change. Less experienced farmers particularly rely on peer social support for advice and farmer discussion groups are the most common source of information on herd health management [19]. These findings informed Intervention E which aims to provide farmers with opportunities to observe other farmers who are similar to them performing target behaviours (e.g., enacting specific animal health management practices), within a supportive environment. Intervention E was well supported: 77% of respondents (*n* = 23) rated it as a “high” or “essential” priority.

Farmers are more likely to trust messages from other farmers due to authenticity of experience and physically seeing the outcomes of changed practices on others’ farms [36]. Others modelling a behaviour can be a very effective method for behaviour change, particularly if the individual sees the ‘model’ receiving reinforcement for the behaviour (e.g., a financial reward, improved herd health, encouragement from others) [37]. Tacit-based learning is also valued by farmers: from observing others, farmers can form a picture of how to perform a behaviour and this can serve as a guide for when they decide to perform this behaviour in the future. For self-efficacy and behavioural capability to be improved in a social support setting, a number of principles are important [9]. Key of which is the selection of the ‘model’ farmer, something also highlighted as important by stakeholders. The model farmer should present a ‘coping’ model rather than a ‘mastery’ model where the challenges to behaviour change are acknowledged as well as the successes. The model should be able to show that they are relatable to other farmers [15]. Rather than focusing on a solely positive story, demonstrating the struggles and challenges they faced in making changes on their farm is viewed as a key factor in ensuring authenticity and making sure that other farmers are able to relate to their experience. It would be important to have a network of several different types of model farmers/farms reflecting different farming situations to ensure as wide a reach as possible to different farmer audiences. Initially, model farms would require significant investment, support and coaching to make the required changes on their farm and be in a place confident enough to share their story.

When utilising peer-led learning, there should be a focus on enactive mastery experiences, where the farmer starts with a simple task that they can achieve before increasing the complexity. Stakeholders cautioned there could be a possible risk of unintended negative outcomes if poor practices or examples were shared by farmers, or if knowledge was exchanged that is not underpinned by strong evidence. In this respect, participants highlighted the importance of expert facilitation and/or technical oversight to ensure that the practices and knowledge being shared amongst farmers is reflective of professional advice and best practice. Vets and farm advisors were viewed as key technical advisors. Stakeholders also cautioned that selection of the specific topics and practices discussed needs to be carefully considered to ensure that the practice in question would be appropriate and applicable for a wide range of farms to incorporate on their farms.

Stakeholders recommended leveraging existing platforms to integrate the aforementioned techniques for peer-to-peer social support and modelling. Discussion groups, demonstration farms and farm walks were frequently mentioned as successful ‘tried and tested’ models that could be leveraged for peer modelling, social learning and knowledge exchange in the AMU space specifically. In particular, farm discussion groups have been found to increase technology adoption through social learning [38]. Morgans et al. [10] found that a farmer-led approach to discuss and reduce AMU in facilitated action groups saw an increased awareness of prudent AMU and a particularly significant reduction in the use of highest priority critically important antibiotics. One-to-one facilitated peer mentoring programmes may have the same benefits as discussion groups for those who opt not to attend a larger group.

### Intervention F: support farmers to monitor their AMU

A desk-based review identified benchmarking as a useful strategy in reducing AMU in other countries but also found few quick, easy or incentivised ways exist for farmers to record and analyse their AMU [19, 22]. This desk research highlighted the need for Intervention F to support the farmer to consistently and frequently monitor their AMU and support them to observe and reflect on the feedback from monitoring (e.g., viewing trends in use over time) and consider how the data compares to a particular goal they may have set for themselves. Intervention F was moderately well supported: 67% of stakeholders (*n* = 20) rated this intervention as a “high” or “essential” priority.

This intervention is grounded in the behavioural science of self-monitoring (or benchmarking), which draws individual attention to one’s current behaviour, identifies areas for improvement and helps keep people on track to achieve goals [9]. Self-monitoring has been used in countless areas to evoke behaviour change; for example, a person using a pedometer and noting their daily number of steps to increase activity. It is worth noting that monitoring as a behaviour change technique is intended to support farmers to make changes that they are intrinsically motivated to do [39]. When monitoring AMU to achieve behaviour change, the most important thing to consider in choosing a method for monitoring AMU, is that it involves a conscious input of information by the farmer, and it offers the ability to download the data for analysis and feedback. This intervention can support farmers to keep track of their AMU and spot patterns, provide positive reinforcement for a behaviour leading to reduced use and sustain the intrinsic motivation of farmers to maintain behaviour change.

Current methods used for monitoring AMU include: farm treatment records; apps for use on smartphones / tablets; excel sheets; handwritten records; collecting empty medicine containers in a bin; veterinary prescription records; E-medicines books. In deploying monitoring as a behaviour change intervention, stakeholders highlighted the existence of current commercial providers of AMU monitoring apps already providing this service to farmers. They warned against double entry across systems and the need to streamline the process at a national standardised level. Stakeholders strongly cautioned against individual interventions that would duplicate any national system for objective measurement and benchmarking of AMU as set out by incoming EU regulations. Stakeholders advised against isolated monitoring interventions, arguing that the introduction of multiple approaches and tools from different providers would negate the benefits that would come from a single centrally coordinated and integrated system that could support accurate national benchmarking. However, some highlighted that despite such requirements for farmers to monitor AMU, and despite tools and technologies available to support monitoring, including apps, there is currently low adherence and poor recording of AMU by farmers. Whilst many supported the principle of monitoring, they felt it would only be effective if farmers were willing to engage in monitoring and engage in it consistently and accurately. The framing and introduction of a monitoring-based intervention to the farming community thus requires important consideration. Stakeholders strongly advised that whatever system is used to implement AMU recording on farms needs to be user friendly. Digital tools and apps were mentioned as useful for on-farm data recording, enabling a more efficient process for farmers, and an automated approach to analysing and displaying trends and patterns. However, care was advised that not all farmers would be digitally comfortable in using such technologies and may have data-sharing concerns. Stakeholders advised a user-centred approach to development to ensure ease and acceptability of technology use.

### Intervention G: develop a supportive community for vets to champion good antimicrobial stewardship

The empirical research found that social norms and peer-to-peer influence were important for vets making changes to AMU, with more experienced vets influencing younger vets’ treatment plans but younger vets increasingly becoming a source of advice on preventive management practices for older vets [4]. Overall, vets were supportive of more preventive measures to combat problematic AMU [19]. These findings highlight how colleagues within a vet’s close circle can persuade and support the vet to engage in more responsible AMU. This informed Intervention G, which proposes utilising peer networks and veterinary practices to start, and support initiatives related to antimicrobial stewardship (AMS). Intervention G was moderately supported: 52% of respondents (*n* = 15) rated this intervention as a “high” or “essential” priority.

Research carried out in Wales successfully forged AMS ‘Champions’ and communities of practice through face-to-face and educational online activities [40]. By initiating tailored AMS interventions within individual practices, Intervention G creates a space for vets to problem-share, address mutual challenges, and learn about feasible alternatives and the latest AMS science and practice. Vets, through their ‘prescribing power’, and their position as a trusted decision-maker for the farmer, are viewed to be in a unique position to influence AMU in farming. Creating a forum for vets to discuss AMU and share experiences and knowledge within their own practices was viewed as positive and particularly important in preparation for the incoming regulatory changes. Stakeholders felt that overall, most governmental, and veterinary representative bodies and organisations should be supportive of this type of intervention, particularly as it would show commitment to change within the community and help bring about a cultural shift in how antibiotics are viewed and used. However, lack of veterinary motivation and uptake was viewed as a potential challenge and stakeholders cautioned the consequences that could arise if some but not all practices made an effort to engage, citing competition between vets as a barrier. Commercial pressure was cited as a challenge, given sales of AMU is a source of monetary income for vets and practices. Stakeholders discussed the need for a cultural shift towards prevention rather than cure, whereby proactive advice would be prioritised over reactive prescription. Different methods for encouraging motivation of vets to participate in this intervention were proposed including a cost incentive for the vet to participate and to compensate their time. Learning the lessons from, and promoting the success of, strategies in other countries (e.g., *RCVS Farm Vet Champion Project*; *Antibiotic Guardian*) was viewed as helpful. The use of benchmarking for vets/practices could be a useful driver for engagement, but this approach could encounter opposition and be counter-productive if vets became demotivated if labelled as ‘under-performing’. Tapping into CPD requirements and offering credits to those taking part in for example, champion training, was also suggested as an avenue for incentivising vets. Linked to this was the concept of a quality assurance model, whereby participating practices could be awarded a ‘badge of honour’ or certified label indicating their commitment and participation to the initiative. To overcome potential commercial concerns, clear boundaries should be established about what can and cannot be discussed and commercially sensitive information or proprietary approaches referenced and credit given where due.

Stakeholders cautioned the need to ensure that the intervention would not be viewed as promoting an authoritarian or exclusionary approach. When the vets transition to advising actions on the farm, they should make these decisions in collaboration with others such as the farm advisor and the farmer. This would ensure collectively acceptable decisions on the farm and avoid potential client backlash / loss of business for the vet. It would also leverage ongoing actions being taken by these actors and ensure that AMU is not viewed as the sole responsibility of the vet. Stakeholders also suggested extension of the intervention itself to other key gatekeepers such as farm advisors. Having the endorsement of veterinary organisations was viewed as important for ensuring community credibility and bringing about larger required cultural changes associated with competitive pressures. Logistically, these organisations were viewed as well positioned to support important elements of this intervention including information provision, resource development, technical oversight, delivery of training, and the setting up and maintenance of an AMS network.

### Summary

Reducing AMU in agriculture requires a focus on behaviour change [8]. The 2022 animal health legislation will require a shift in how antimicrobials are used in some areas of agriculture. Yet this shift can only be achieved through understanding farmers’ and vets’ needs and helping them to address any necessary changes at farm level. The agri-food community should recognise the varied levels of preparedness across farmers and vets to navigate the incoming regulations and identify resources that will support them through this major transition. The current findings highlight the importance of answering questions such as: how much, and where, antimicrobials are being used; why antimicrobials are being used, and how to target those drivers. This offers a new approach to rethinking how best to tackle AMR in the animal health sector, a challenge that has proven difficult to address to date [41]. Rather than focusing on the delivery of the scientific evidence to the farming sector, and expecting change to happen, the current study highlights the need for a greater focus on supporting change to happen through a systematic approach to behaviour change, and bottom-up interventions which work with actors in the farming community, in particular, farmers, vets and farm advisors.

Widespread and long-lasting behaviour change will only come about when we address the multi-faceted individual, interpersonal, organisational, financial and societal-level determinants shaping antimicrobial use on farms [15]. From a behavioural science perspective, to bring about long-lasting behaviour change, in lieu of a solely restrictive approach, it is necessary to enable farmers to adopt new approaches to farm management that will inevitably have knock-on effects for the overall sustainability of agriculture [10, 12]. While acknowledging the role that top-down regulatory approaches can play in behaviour change, our research focused largely on those bottom-up behaviour change interventions which can be implemented by key stakeholders in the sector to tackle capability (e.g. increasing knowledge and awareness), motivation (e.g. tackling attitudes and beliefs) and opportunity (e.g. provision of social support). This aligns with the approaches most favoured by farmers themselves – in the farmer survey carried out as part of the AMU Project, farmers rated “new policies and regulations to restrict antibiotic use” on farms as the least favoured intervention approach [19]. This signals the need to embed bottom-up behaviour change interventions that increase farmers’ knowledge, motivation and opportunity for change alongside regulations that would enforce such change.

## Conclusions

The current study outlines seven bottom-up behaviour change interventions that can be taken, adapted, and put into practice by the agri-food community to support good animal health practices and responsible AMU on farms. The next step for these interventions is to carry out field trials and controlled pilots to evaluate the level of impact they can achieve in the applied setting. Where possible, pilot interventions should take a nested Community of Practice multi-actor approach to their implementation. The approach undertaken for intervention design in this study combines the theoretical, the empirical, and the practical; the concepts for the seven behaviour change interventions are grounded in behavioural science and psychological theory, while their content and delivery are informed by stakeholder suggestions and feedback. This approach ensures that both scientific and tacit knowledge is integrated to best effect [16]. By combining inter-disciplinary research with a co-design process, resultant ideas are a synthesis of empirical research and local and practical knowledge provided by key stakeholders, leading to greater legitimacy and uptake [18]. The collaborative nature of a multi-actor approach can help to engender a sense of ownership, transparency and inclusiveness amongst key actors in the development and implementation of intervention options that will be key to their success.

The current study offers a novel approach to designing behaviour change interventions for AMU in agriculture. Strengths of the study include a systematic, evidence-based approach to behaviour change intervention grounded in behavioural science and a participatory approach. A caveat of the current study is that the interventions suggested have not been tested and future research is required to pilot and evaluate the interventions.

## Supplementary Information

**Additional file 1:** **Appendix 1. **Stakeholder Maps.

## Data Availability

The datasets used and/or analysed during the current study are available from the corresponding author on reasonable request.
