# Supplementary material for: Behaviour change interventions for responsible antimicrobial use on farms
Source: Ir Vet J. 2023 Apr 3;76:8. doi: 10.1186/s13620-023-00236-x (PMC10068206; doi:10.1186/s13620-023-00236-x)
Supplement: Supplementary file 1 — Additional file 1: Appendix 1. Stakeholder Maps. [file 13620_2023_236_MOESM1_ESM.docx]

Additional File 1 / Appendix 1: Stakeholder Maps


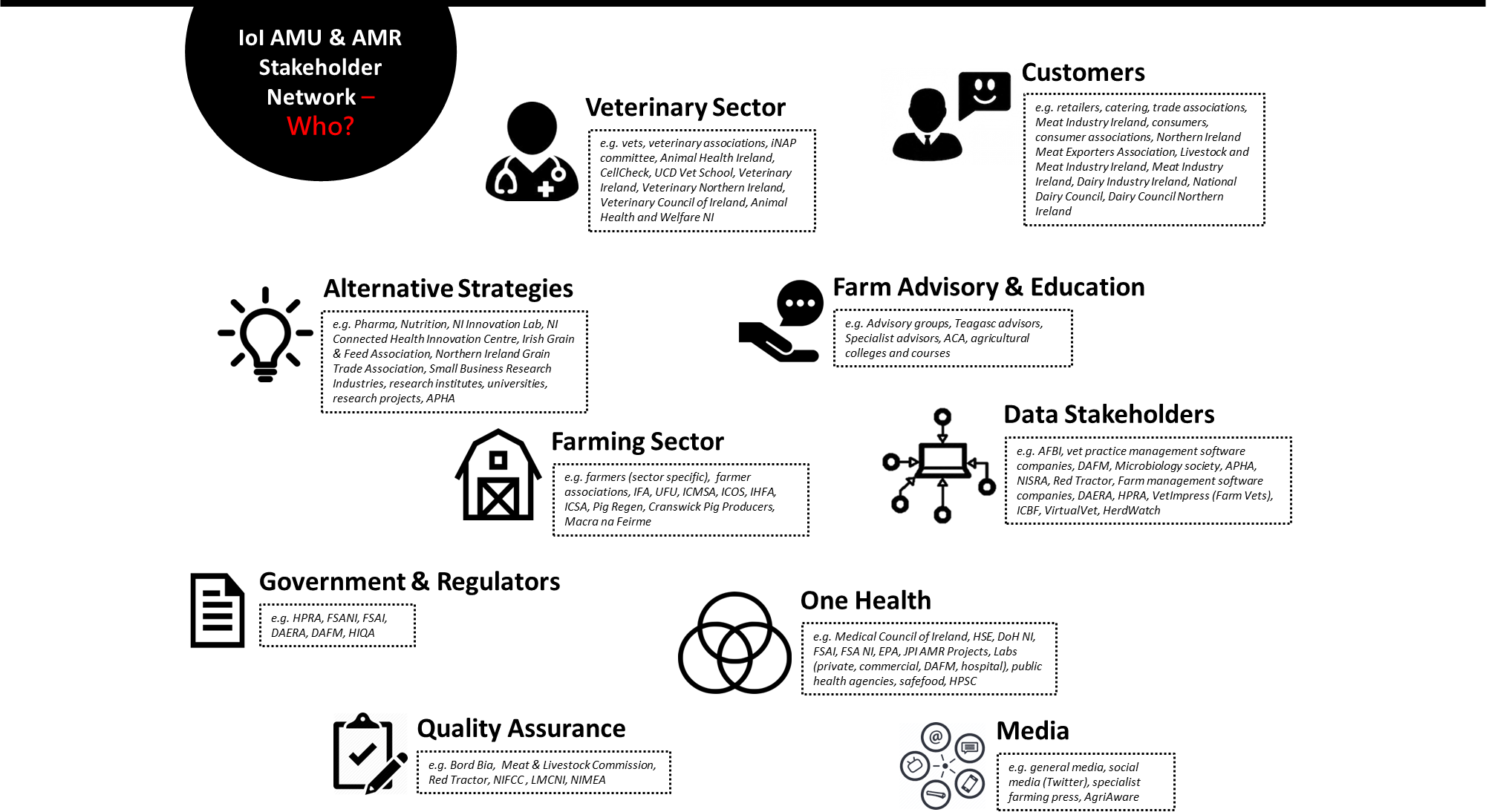


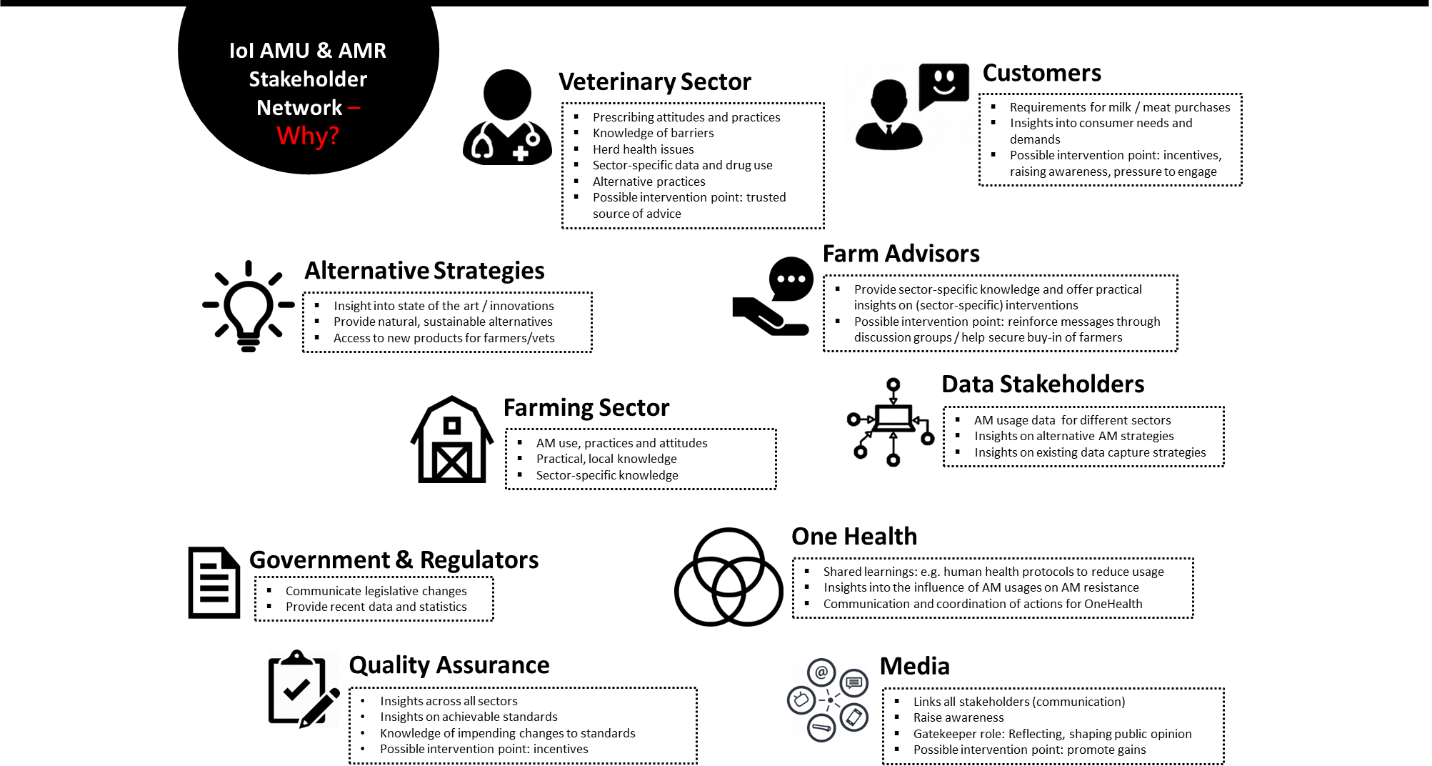


Figure A1: Stakeholder Maps produced through participatory workshops identifying relevant stakeholders interested in AMU and AMR on the island of Ireland
